# Polyunsaturated fatty acids in fish tissues more closely resemble algal than terrestrial diet sources

**DOI:** 10.1007/s10750-020-04445-1

**Published:** 2020-11-16

**Authors:** Nadine Ebm, Fen Guo, Michael T. Brett, Stuart E. Bunn, Martin J. Kainz

**Affiliations:** 1 WasserCluster Lunz – Inter-university Center for Aquatic Ecosystem Studies, 3293 Lunz Am See, Austria; 2 Functional and Evolutionary Ecology, Faculty of Life Sciences, University of Vienna, 1090 Vienna, Austria; 3 Simon F.S. Li Marine Science Laboratory, School of Life Sciences, The Chinese University of Hong Kong, Hong Kong, China; 4 Department of Civil and Environmental Engineering, University of Washington, Seattle, WA 98195 USA; 5 Australian Rivers Institute, Griffith University, Nathan, QLD 4111 Australia; 6 Department for Biomedical Research, Danube University Krems, Krems an der Donau, Austria

**Keywords:** Stream food webs, Food quality, Headwaters, Fish brain, Fish eyes, Docosahexaenoic acid

## Abstract

The River Continuum Concept implies that consumers in headwater streams have greater dietary access to terrestrial basal resources, but recent studies have highlighted the dietary importance of high-quality algae. Algae provide consumers with physiologically important omega-3 (n-3) polyunsaturated fatty acids (PUFA), particularly eicosapentaenoic acid (EPA). However, terrestrial plants and most benthic stream algae lack the long-chain (LC) n-3 PUFA docosahexaenoic acid (DHA, 22:6n-3), which is essential for neural development in fish and other vertebrates. We sampled subalpine streams to investigate how the PUFA composition of neural (brain and eyes), muscle, and liver tissues of freshwater fish is related to their potential diets (macroinvertebrates, epilithon, fresh and conditioned terrestrial leaves). The PUFA composition of consumers was more similar to epilithon than to terrestrial leaves. Storage lipids of eyes most closely resembled dietary PUFA (aquatic invertebrates and algae). However, DHA and arachidonic acid (ARA, 20:4n-6) were not directly available in the diet but abundant in organs. This implies that algal PUFA were selectively retained or were produced internally via enzymatic PUFA conversion by aquatic consumers. This field study demonstrates the nutritional importance of algal PUFA for neural organs in aquatic consumers of headwater regions.

**Electronic supplementary material:**

The online version of this article (10.1007/s10750-020-04445-1) contains supplementary material, which is available to authorized users.

## Introduction

At the base of aquatic food webs, primary producers, e.g., microalgae, provide consumers with dietary nutrients, including essential polyunsaturated fatty acids (PUFA), that are crucial constituents of cell membranes (e.g., phospholipids). A steady supply of dietary PUFA to aquatic consumers, particularly omega-3 (n-3) long-chain (LC) PUFA, such as eicosapentaenoic acid (EPA; 20:5n-3) and docosahexaenoic acid (DHA; 22:6n-3), is critical as these fatty acids (FA) support somatic growth and reproduction (Müller-Navarra et al., 2000; Brett et al., 2009). However, most aquatic consumers have limited abilities to synthesize EPA and DHA and thus have to take up these biomolecules directly from their diets (Brett & Müller-Navarra, 1997; Arts et al., 2009; Torres-Ruiz et al., 2010). Vascular plants synthesize the short-chain PUFA: alpha-linolenic acid (ALA; 18:3n-3) and the n-6 PUFA linoleic acid (LA, 18:2n-6), but generally lack EPA and DHA (Brett et al., 2009; but see Napier, 2006). Although epilithic algae (especially diatoms) in freshwater ecosystems are comparatively rich in EPA, they are low in DHA (Brett et al., 2009; Hixson et al., 2015; Guo et al., 2018). However, DHA is essential for all vertebrates including freshwater fishes (Ahlgren et al., 1994; Guo et al., 2017) where it is associated with high membrane fluidity (e.g., signal transduction, neurotransmission or hormone regulation, Farkas et al., 2000), anti-inflammatory and—oxidative effects (e.g., neuroprotection, Bazan, 2005), proper neural development (cognitive performance, Lund et al., 2014), and sensory functioning (visual acuity, Bell et al., 1995).

In contrast to marine fish (Agaba et al., 2005; Tocher et al., 2006; Mohd-Yusof et al., 2010), previous studies have reported that enzymatic conversions to LC-PUFA are functional in some freshwater fish (e.g., *Oncorhynchus mykiss* (Walbaum, 1792), *Salvelinus alpinus* (Linnaeus, 1758), Buzzi et al., 1996; Tocher et al., 2001; Murray et al., 2014). These fish are able to transform dietary ALA via stearidonic acid (SDA) and EPA to DHA, but direct incorporation of dietary DHA is preferred by fish as it reduces the more energy-demanding endogenous production pathway. However, in contrast to such experimental studies, little is known about the PUFA pathways to and within fish in natural ecosystems deprived in DHA (Gladyshev et al., 2018), and especially how fish in streams acquire their PUFA and subsequently allocate these FA to different organs, including neural organs (Guo et al., 2017).

Neural organs, e.g., brain and eyes, are rich in DHA (Mourente & Tocher, 1998; Farkas et al., 2000; Stoknes et al., 2004), which is key for proper functioning of these organs and eventually optimal fish physiological performance. Common stream fish in alpine headwaters (e.g., salmonids and European bullhead) are visual predators that prey mostly on moving or drifting invertebrates oftentimes at night (Elliott, 1973; Mills & Mann, 1983). A reduced visual performance (Bell et al., 1995) or learning ability (Lund et al., 2014) of fish due to LC-PUFA-poor diets can reduce feeding success, predator avoidance (e.g., escape latency, swimming speed) and other behavioral traits, e.g., schooling behavior (Ishizaki et al., 2001). Despite the fact that neural organs have the highest lipid contents and are the most energy-demanding tissues in animals, our current knowledge about energy flows in aquatic food webs is primarily derived from studies on fish muscle and liver tissues (Volk & Kiffney, 2012; Kainz et al., 2017; Sushchik et al., 2018). Previous research indicated that FA in neutral lipids (NL) of fish muscles, such as triacylglycerols (TAG), reflect those from the diet, while FA in the polar lipids (PL) of fish muscles are driven by taxonomy (Iverson 2009; Sushchik et al., 2020). Total lipids and TAG contents vary among fish organs (Tocher & Harvie, 1988; Budge et al., 2006; Hong et al., 2014) and thus different organs may vary in correspondence to dietary FA.

Most vertebrate and fish brains consist mainly of PL (Soengas & Aldegunde, 2002), which are rich in DHA (Tocher & Harvie, 1988; Iverson, 2009), making them indispensable for cell functionality and relatively stable to dietary lipid intake. In contrast, fish eyes are rich in TAG (Mourente et al., 1991; Geurden et al., 1998; Stoknes et al., 2004) and thus eye lipids may reflect dietary PUFA sources better than brain lipids (Brodtkorb et al., 1997). Bell et al. (1995) demonstrated that DHA-poor diets resulted in DHA-deficit retina membranes with impaired vision under natural light intensities. It is thus important to understand how lipid classes in neural organs are affected by dietary FA. However, this has not been tested yet for stream fish which receive very little DHA directly from organisms at the base of the food chain.

Motivated by the ambiguities outlined above, we designed a field study to investigate the PUFA distribution in freshwater fish and their potential diet sources in subalpine streams. We compared the PUFA composition of various fish organs, i.e., muscle, liver, brain, and eyes, with PUFA of potential diet sources (terrestrial plants, benthic algae and macroinvertebrates), and also explored the FA distribution of salmonid brain and eyes in an effort to discern how different lipid classes (NL versus PL) of these neural organs are affected by dietary PUFA sources. We tested the hypotheses that, (1) the PUFA composition of stream consumers (macroinvertebrates and fish) is generally more similar to epilithon than to the leaves of terrestrial vascular plants, and, (2) fish eyes have a greater similarity with dietary PUFA than fish brains due to differences in lipid class composition.

## Methods

### Study streams

We sampled 17 sites in 9 oligotrophic streams (1st-5th order, total length ~ 42 km) within the headwater region (‘Weiße Ois’) of the subalpine River Ybbs catchment, Austria (Online resource 1). The study catchment (550–1000 m.a.s.l.) has a nivo-pluvial flow regime that drains 254 km^2^ and is characterized by glaciokarst and dolomite (82%), with forests (82%) and alpine meadows (11%) the predominant land cover forms (Besemer et al., 2013). The river substratum is dominated by gravel and cobble with intense bed-load transport during peak flows. The typical fish species in these study streams are Brown trout (*Salmo trutta fario* Linnaeus, 1758), European bullhead (*Cottus gobio* Linnaeus, 1758), European grayling (*Thymallus thymallus* Linnaeus, 1758) and Rainbow trout (*Oncorhynchus mykiss*). Stocking of Brown and Rainbow trout is reported but only at the downstream study sites (YKL, YLG, and YGL). Chemical parameter (N-NO_3_, N-NH_4_, P-PO_4_), soluble reactive phosphorus (SRP), and dissolved organic matter (DOM) concentrations in these study streams were reported in a previous study (Guo et al., 2018).

### Sample collection

Potential dietary resources for fishes, including epilithon, conditioned terrestrial leaves, and aquatic invertebrates were collected in October 2016. Conditioned leaves (submerged, senescent, and brownish leaves of various vascular plant species) were collected from streams). Epilithon from each of the sampling sites was transferred into sample containers from small cobbles using soft brushes and invertebrates were picked from rocks. The taxonomic composition of epilithon and periphyton (biofilm on submerged leaves) was not examined because we were primarily interested in their dietary and biochemical composition (Guo et al., 2018). Fresh leaves were picked directly from riparian vegetation in summer (July 2016). These samples included angiosperms (*Acer*, *Alnus*, *Corylus avellana* L., *Fagus sylvatica* L., *Hedera helix* L., *Phragmites*, *Salix* and gymnosperms (*Picea* and *Pinus*) and each plant species was analyzed separately.

Macroinvertebrates were identified to the genus level using a stereomicroscope and pooled by species for lipid analyses. Macroinvertebrate samples included Ephemeroptera (*Baetis*, *Ecdyonurus*, *Rhithrogena*), Plecoptera (*Leuctra*, *Nemoura*, *Perla*, *Perlodes*, *Isoperla*), Trichoptera (*Allogamus*, Odontoceridae, *Plectronemia*, *Potamophylax, Rhyacophila*, *Hydropsyche*), Platyhelminthes and the amphipod *Gammarus*.

Fish capture and collection for this research was permitted by the local fisheries authorities for these study streams. Individuals of three fish (*O. mykiss*, *S. trutta*, and *C. gobio*) were collected by electrofishing in November 2016, anesthetized and subsequently killed in accordance with the Federal Act on the Protection of Animals, Austria (http://www.ris.bka.gv.at). Fish total body length (mm) and weight (g) were recorded (Online Resource 2). No fish were found at the Schelchen (SCH) sampling site. Invertebrate samples were kept on ice in a portable cooling box and stored at cryogenic temperature (− 80°C) in the lab. Fishes were dissected and samples of the dorsal muscle and the entire liver of each specimen were collected. Dermis and subcutaneous tissue of each muscle sample were removed and excluded from further biochemical analyses. Entire brains and eyeballs together, with the optic nerve, were removed from each fish. In some cases, in particular for European bullhead, some eye or brain samples had to be pooled prior to lipid extraction (1–5 individuals matched for similar size from each sampling site) to obtain sufficient biomass for lipid analysis. All samples were stored on ice during dissection, placed into Eppendorf tubes (brain and eyes) or scintillation vials (muscle and liver) and stored at cryogenic temperature (− 80°C) until lyophilization (Virtis Genesis™ freeze dryer).

### Lipid analyses

#### Total lipid extraction

Lipids and their FA were analyzed as described by Guo et al. (2015). Briefly, total lipids from freeze-dried (i.e., all lipids and FA were reported as dry weight; DW) and homogenized samples (fresh and conditioned terrestrial leaves: ~ 50 mg, epilithon: ~ 10 mg, invertebrates: ~ 5 to 7 mg, brain and eyes: ~ 5 mg, liver: ~ 10 to 15 mg, muscle: ~ 15 to 20 mg) were dissolved in ice-cold chloroform (2 mL) and stored under N_2_ atmosphere over night at − 80°C to improve lipid extraction efficiency. Samples were then further extracted in chloroform–methanol (2:1) and NaCl (0.8 mL; salt wash), vortexed and sonicated, and subsequently analyzed gravimetrically in pre-weighed tin capsules (total lipid content determination).

#### Lipid class separation

Only salmonids (*S. trutta* and *O. mykiss*) were used for the analysis of FA in lipid classes in neural organs because of limited material for lipid class separation for European bullhead brain and eye samples. Lipid extracts of selected salmonid brain and eye samples were separated into lipid classes by thin-layer chromatography (TLC). Mass ratios of lipid extracts were adjusted after gravimetry with chloroform to obtain similar lipid amounts (150–200 µg) in the volume (50 µL) applied to the TLC plates for salmonid brain and eye samples only. Polar (membrane lipids, PL) and neutral lipids (storage lipids, NL) were separated by one-dimensional TLC on 10 × 10 cm silica gel plates (Merck TLC silica gel 60) using a hexane:diethylether:methanol:formic acid (90:20:3:2, v/v/v/v) solvent solution. After development, plates were sprayed with 0.05% (wt/vol) 8-anilino-4-naphthalenesulfonic acid in methanol and viewed under UV light to detect lipid fractions (Online resource 3). An internal standard (5 µL; non-adecanoic acid in chloroform; 4 mg mL) was added to each lipid fraction before individual lipid fractions were scraped from the TLC plates and transferred into separate solvent-rinsed vials.

#### Fatty acid methylation and analysis

Fatty acids from total lipid extracts and after lipid class separation were derivatized to fatty acid methyl esters (FAME) in a H_2_SO_4_ methanol solution for 16 h at 50°C. All FAME were stored at − 80°C until being separated using gas chromatography (THERMO™ Trace GC) and detected using flame ionization detection (FID). FAME were separated by a Supelco™ SP-2560 column (100 m, 25 mm i.d., 0.2 µm film thickness), identified by comparison to the retention times of known standards (37-component FAME Mix, Supelco 47885-U; Bacterial Acid Methyl Ester Mix, Supelco 47080-U). The FAME concentrations were quantified using calibration curves based on known standard concentrations. All FAME analyses were replicated within the study design (e.g., 5 epilithon samples per sample site). Results for each FAME were reported as relative values (% of total FAME).

#### Data analysis

Before multivariate analysis, all PUFA (%) data were arcsine-square-root-transformed for normal distribution approximation and variance stabilization. Multivariate outliers were removed by the minimum volume ellipsoid-based robust distance (Mahalanobis, *R* package “mvoutlier”, Filzmoser & Gschwandtner, 2018).

Six individual PUFA (LA, ARA, ALA, SDA, EPA, and DHA) were selected for non-metric multidimensional scaling (nMDS) to ordinate the PUFA composition among fish and their food sources (leaves, algae and invertebrates) in two-dimensional space as a function of rank-order dissimilarity (Bray–Curtis). Analysis of similarity (ANOSIM, *R* package “vegan”, Oksanen et al., 2019) with 999 permutations was then used to assess similarities in the PUFA composition between food sources (leaves, algae and invertebrates), fish or their organs by ANOSIM global R value. This value ranges from − 1 to + 1. A global *R* statistic close to 1 indicates that the pair (or a set of samples) is different; an R statistic close to 0 implies that the pair is similar. In greater detail, a global *R* value of < 0.25 indicates that groups are hardly separated, *R* < 0.5 shows that groups differ but with some overlapping, and *R* > 0.75 implies groups are very well separated (Jaschinski et al., 2011). Although not commonly reported, negative values can occur and indicate greater dissimilarity within a group than among groups (Chapman & Underwood, 1999). In addition, a regression tree (R package “tree”, Ripley, 2019) was performed with untransformed proportion data (Online resource 4) to explore patterns between individual PUFA and organisms at different trophic levels and among fish organs or lipid classes. The acceptable level of misclassification was set at < 0.30. The significance threshold for all data analyses was set at *P* < 0.05. For visualization of differences in the PUFA composition between salmonid brain and eye lipid classes, abundant FA other than PUFA: i.e., saturated FA (14:0 = myristic acid, 16:0 = palmitic acid, 18:0 = stearic acid) and monounsaturated FA, oleic acid (18:1n-9) and the diatom biomarker, palmitoleic acid (16:1n-7), were added to the previously selected 6 PUFA to better interpret the position of samples in the ordination space. For all presented ordination plots stress values < 0.1 were achieved.

## Results

### PUFA composition

**Fig. 1:**
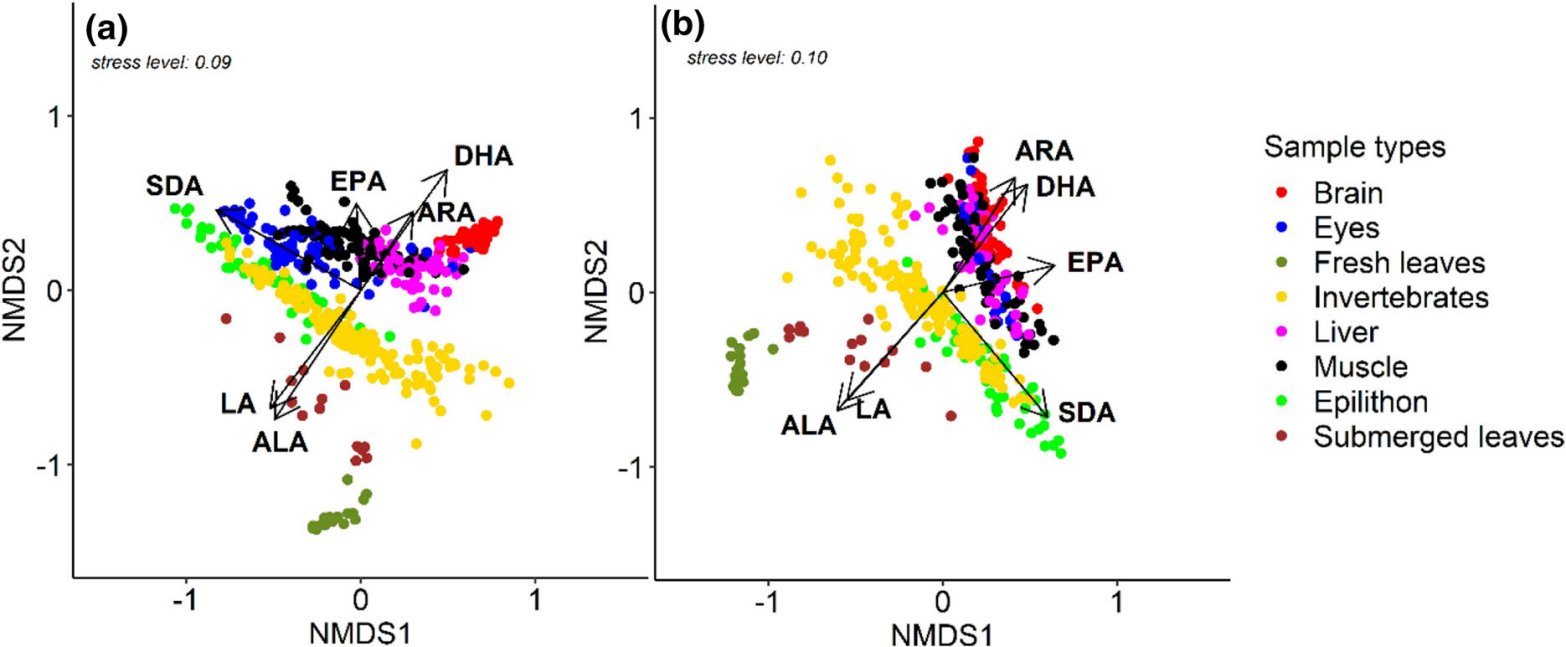
Non-metric multidimensional scaling (nMDS) of arcsine-square-root-transformed PUFA (% of total FAME) of basal resources, benthic invertebrates and **a** salmonids (*S. trutta* and *O. mykiss*) and **b** European Bullhead (*C. gobio*). *ALA* alpha-linolenic acid, *SDA* stearidonic acid, *EPA* eicosapentaenoic acid, *DHA* docosahexaenoic acid, *LA* linoleic acid, *ARA* arachidonic acid

The PUFA composition of muscle, liver, brain and eye samples in sedentary (European bullhead) and potentially migratory (Brown and Rainbow trout) fishes differed from that of their potential food sources (macroinvertebrates, epilithon, fresh and conditioned terrestrial leaves) (Fig. 1, Table 1). Basal resources (epilithon, fresh and conditioned leaves) in streams differed significantly from each other in their PUFA composition (ANOSIM; *R* = 0.84, *P *< 0.001, *n* = 84). Fresh leaves contained predominantly the short-chain PUFA ALA and LA, whereas conditioned leaves additionally contained traces of SDA and EPA (Table 2). Compared to leaves, epilithon and benthic invertebrates contained high levels of EPA and traces of DHA, which led to a further split in the classification tree between the latter two (online resource 4). Further, epilithon samples contained the highest levels of the diatom biomarker 16:1n-7 (Table 2). Fish differed mostly from their potential resources (macroinvertebrates, epilithon, fresh and conditioned leaves) by their high DHA content, particularly in brain tissue. Nonetheless, the PUFA composition of consumers (benthic macroinvertebrates and fish) was more similar to algal than to terrestrial resources (fresh and conditioned leaves, Table 3).

**Table 1:** Key statistical parameters of non-metric multidimensional scaling (nMDS) of arcsine-square-root-transformed PUFA (% of total FAME) of basal resources, benthic invertebrates and (a) salmonids (*S. trutta* and *O. mykiss*) and (b) European Bullhead (*C. gobio*)

	FA	nMDS scores		*r*	*P*
		Axis 1	Axis 2		
Salmonids	EPA	− 0.05161	0.99867	0.50	< 0.001
	ARA	0.5554	0.83159	0.54	< 0.001
	DHA	0.5837	0.81197	0.85	< 0.001
	LA	− 0.60997	− 0.79243	0.85	< 0.001
	ALA	− 0.55755	− 0.83014	0.89	< 0.001
	SDA	− 0.87338	0.48704	0.95	< 0.001
European bullhead	EPA	0.97165	0.23644	0.66	< 0.001
	ARA	0.5331	0.84605	0.78	< 0.001
	DHA	0.61576	0.78794	0.79	< 0.001
	LA	− 0.66207	− 0.74944	0.83	< 0.001
	ALA	− 0.66585	− 0.74608	0.91	< 0.001
	SDA	0.64262	− 0.76619	0.93	< 0.001

Vectors (fatty acids, FA) are sorted ascending according to its correlation coefficient (*r*). *ALA* alpha-linolenic acid, *SDA* stearidonic acid, *EPA* eicosapentaenoic acid, *DHA* docosahexaenoic acid, *LA* linoleic acid, *ARA* arachidonic acid

**Table 2:** Individual PUFA (% of total FAME) among different food-web components in streams (mean ± 1 standard deviation)

Samples		ALA	SDA	LA	EPA	ARA	DHA	16:1n-7	*n*
Terrestrial resources	Fresh leaves	37 ± 20	0 ± 0	16 ± 8	0 ± 0	0 ± 0	0 ± 0	1 ± 0	22
	Conditioned leaves	31 ± 4	1 ± 1	10 ± 1	1 ± 1	0 ± 0	0 ± 0	1 ± 1	14
Aquatic resources	Epilithon	9 ± 3	3 ± 2	7 ± 2	12 ± 5	1 ± 0	1 ± 0	15 ± 5	48
	Invertebrates	10 ± 4	1 ± 1	6 ± 3	14 ± 6	1 ± 1	0 ± 0	8 ± 4	146
European bullhead	Brain	2 ± 2	1 ± 0	1 ± 1	11 ± 3	4 ± 1	25 ± 4	5 ± 1	37
	Eyes	6 ± 4	1 ± 1	3 ± 1	8 ± 2	2 ± 1	19 ± 7	7 ± 3	24
	Liver	4 ± 2	1 ± 1	3 ± 1	12 ± 4	5 ± 2	19 ± 6	5 ± 2	48
	Muscle	5 ± 3	1 ± 1	3 ± 1	18 ± 4	3 ± 2	15 ± 5	5 ± 3	57
Salmonids	Brain	1 ± 0	0 ± 0	1 ± 0	8 ± 1	1 ± 0	32 ± 5	2 ± 1	70
	Eyes	10 ± 4	2 ± 1	5 ± 2	7 ± 2	1 ± 1	11 ± 7	12 ± 4	64
	Liver	4 ± 1	1 ± 0	3 ± 1	10 ± 3	4 ± 2	26 ± 7	4 ± 1	60
	Muscle	7 ± 3	1 ± 1	3 ± 1	13 ± 3	2 ± 1	24 ± 6	5 ± 2	90

*ALA* alpha-linolenic acid, *SDA* stearidonic acid, *EPA* eicosapentaenoic acid, *DHA* docosahexaenoic acid, *LA* linoleic acid, *ARA* arachidonic acid, *n* sample size

**Table 3:** Pairwise similarity (ANOSIM; R) of FA compositions among basal resources, benthic invertebrates and fish (brain, eyes, liver and muscle)

Sample types	Basal resources			Invertebrates
	Fresh leaves	Conditioned leaves	Epilithon	
Fish (all organs)	0.9974*** *n* = 472	0.8171*** *n* = 464	0.475*** *n* = 498	0.4891*** *n* = 596
Invertebrates	0.8899*** *n* = 168	0.396*** *n* = 160	0.235*** *n* = 194	–
European bullhead				
Brain	1*** *n* = 59	0.9932*** *n* = 51	0.8148*** *n* = 85	0.5109*** *n* = 183
Eyes	1*** *n* = 92	0.9278*** *n* = 38	0.555*** *n* = 72	0.3379*** *n* = 72
Liver	1*** *n* = 70	0.9541*** *n* = 62	0.617*** *n* = 96	0.3214*** *n* = 96
Muscle	1*** *n* = 79	0.9103*** *n* = 71	0.5438*** *n* = 105	0.3195*** *n* = 105
Salmonids				
Brain	1*** *n* = 92	1*** *n* = 92	0.9923*** *n* = 118	0.8398*** *n* = 216
Eyes	0.9989** *n* = 86	0.8222*** *n* = 78	0.1869*** *n* = 112	0.3901*** *n* = 210
Liver	1*** *n* = 82	0.9973*** *n* = 74	0.8943*** *n* = 108	0.4348*** *n* = 206
Muscle	1*** *n* = 112	0.9607*** *n* = 104	0.5857*** *n* = 138	0.3974*** *n* = 236

Higher Global *R* values indicate higher dissimilarity between two or more groups. Asterisks indicate significance levels: ***P ≤ 0.001, **P ≤ 0.01, *P ≤ 0.05

The organs of both salmonid taxa (*S. trutta* and *O. mykiss*) did not differ fundamentally in their PUFA composition and will subsequently be referred to as salmonids. The PUFA composition of salmonid organs grouped distinctly from each other (ANOSIM: *R* = 0.68, *P *< 0.001, *n* = 284), but those of European bullhead overlapped extensively (ANOSIM *R* = 0.09, *P* = 0.002, *n* = 166). However, the FA profiles of the organs still differed from each other in both the salmonids and European bullhead. In both fish taxa, brains had the lowest LA and ALA and the highest DHA content (Table 2). Compared to brains, the eyes contained more LA and ALA, but less EPA than liver and muscle samples, and livers contained the greatest levels of ARA. European bullhead had higher brain ARA and lower liver DHA levels than in salmonids, but muscle PUFA did not differ between the two fish taxa (ANOSIM R = 0.18, *P *< 0.001, *n* = 147). Brain and eye samples of European bullhead (ANOSIM: *R* = 0.19, *P *= 0.002, *n *= 61) and salmonids (ANOSIM: *R* = 0.93, *P *< 0.001, *n* = 134) were the most dissimilar organs. In both fish taxa, eye PUFA had the highest similarity with dietary PUFA (Table 3) and the highest levels of 16:1n-7 (Table 2).

#### FA composition in lipid classes of fish brain and eyes

**Fig. 2:**
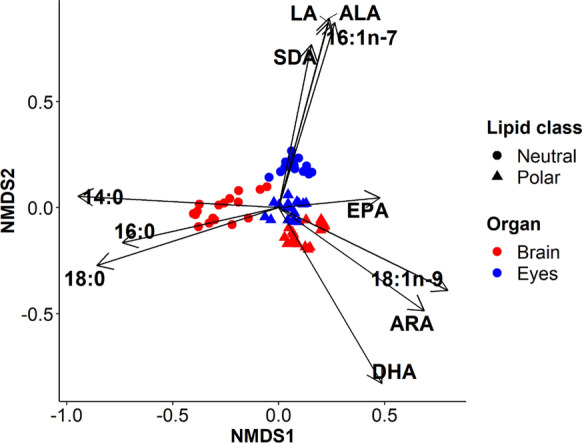
Non-metric multidimensional scaling (nMDS) of arcsine-square-root-transformed fatty acids (% of total FAME) of salmonid (*S. trutta* and *O. mykiss*) polar and neutral lipids from the brain and eyes. 14:0 = myristic acid, 16:0 = palmitic acid, 18:0 = stearic acid, 18:1n-9 = oleic acid, *ALA* alpha-linolenic acid, *SDA* stearidonic acid, *EPA* eicosapentaenoic acid, *DHA* docosahexaenoic acid, *LA* linoleic acid, *ARA* arachidonic acid

Salmonid eyes contained 4 times more NL than PL, but their brains had 5 times more PL than NL (online resource 5). The nMDS (Fig. 2) of the FA in the lipid classes of the salmonid brain and eye samples showed that PL differed significantly from NL in both organs (ANOSIM *R* = 0.89, *P *< 0.001, *n* = 70, Fig. 2). Axis 1 of the nMDS separated brain and eye PL from brain NL on the basis of their LC-PUFA (correlation coefficients: DHA = 0.96, ARA = 0.84, EPA = 0.48), 18:1n-9 (*r* = 0.89) and SAFA ($1 4 : 0$  = 0.95, $1 8 : 0$  = 0.95 $, 1 6 : 0$ = 0.76) content. Axis 2 of the nMDS segregated eye NL from brain and eye PL correlated strongly with C_18_-PUFA (ALA = 0.92, LA = 0.90, SDA = 0.78) and the diatom marker 16:1n-7 (*r *= 0.91).

Brain- and eye PL were more similar (ANOSIM; *R* = 0.70, *P *< 0.001, *n* = 38) to each other than NL of both these organs (ANOSIM; *R* = 0.97, *P *< 0.001, *n* = 32). Generally, PL in both organs differed from NL by having a higher DHA content (Table 4). Brain PL were characterized by very low levels of LA compared to eyes. Neutral lipids of eyes contained higher contents of ARA than brain NL. Finally, NL of eyes had high contents of ALA, LA, SDA and the diatom biomarker (16:1n-7). Additionally, brain NL were rich in saturated fatty acids (SAFA).

**Table 4:** Fatty acid content (% of total FAME) in brain and eye lipid classes of *S. trutta* and *O. mykiss* combined (mean ± 1 standard deviation)

Organ	Lipid class	14:0	16:0	18:0	18:1n-9	16:1n-7	ALA	SDA	LA	ARA	EPA	DHA
Brain	Neutral	22 ± 6	29 ± 5	24 ± 7	5 ± 3	2 ± 2	1 ± 2	0 ± 1	1 ± 1	0 ± 0	1 ± 2	3 ± 3
	Polar	3 ± 2	20 ± 2	9 ± 2	18 ± 3	2 ± 1	1 ± 0	0 ± 0	0 ± 0	1 ± 0	3 ± 4	23 ± 4
Eyes	Neutral	5 ± 3	19 ± 2	6 ± 3	12 ± 3	12 ± 2	10 ± 3	3 ± 2	5 ± 1	1 ± 0	2 ± 4	3 ± 0
	Polar	6 ± 4	23 ± 4	13 ± 4	12 ± 3	6 ± 2	3 ± 1	1 ± 0	2 ± 1	1 ± 0	2 ± 4	15 ± 4

14:0 = myristic acid, 16:0 = palmitic acid, 18:0 = stearic acid, 18:1n-9 = oleic acid, *ALA* alpha-linolenic acid, *SDA* stearidonic acid, *EPA* eicosapentaenoic acid, *DHA* docosahexaenoic acid, *LA* linoleic acid, *ARA* arachidonic acid

## Discussion

Earlier studies on dietary lipid and transfer along freshwater food webs focused on how dietary energy is passed from sources to whole consumers or selected parts thereof, e.g., dorsal muscle tissue of fish (Ballantyne et al., 2003; Kainz et al., 2004, 2017). By looking at selected parts of fish only, inferences about dietary energy transfer are limited to these tissues, and cannot be extended to whole organisms, thus limiting comprehensive assessments. This is the first study that shows the dietary transfer of LC-PUFA from epilithon to stream invertebrates and subsequently to various fish tissues, including neural organs.

At the base of stream food webs, we detected a substantial lack of LC-PUFA in terrestrial leaves (submerged and fresh leaves) compared to high EPA contents in epilithon samples. High EPA levels found in biofilms are an important dietary resource supporting somatic growth and reproduction of aquatic invertebrates (Goedkoop et al., 2007; Lau et al., 2008). Conditioned terrestrial leaves contained only traces of EPA, perhaps because of associated microalgae but, similar to earlier studies (Torres-Ruiz et al., 2007; Guo et al., 2016) still provided food of higher nutritional quality than fresh leaves for invertebrates. Naturally occurring terrestrial vascular plants lack the enzymes necessary to synthesize EPA or other LC-PUFA and are difficult to digest due to their high lignocellulose content (Brett et al., 2017). Consequently, EPA retention from epilithic biofilms is more beneficial instead of energy-demanding internal bioconversion from precursors. Stream invertebrates have a limited innate ability to transform ALA to EPA (‘trophic upgrading’) and thus their PUFA composition mostly resembles dietary PUFA (Torres-Ruiz et al., 2007; Masclaux et al., 2012). The PUFA profiles of benthic invertebrates were more similar to epilithon rather than to leaves, confirming that invertebrates mainly depend on algae (Lau et al., 2009; Guo et al., 2017, 2018). As a result, benthic biofilms colonizing stream rocks with a high content of physiologically required EPA convey nutrients of higher dietary high-quality conducive for consumer growth, than does terrestrial vegetation.

There was an evident mismatch between dietary LC-PUFA (terrestrial leaves, epilithon and aquatic invertebrates) and riverine fish, as previously noted by Guo et al. (2017). Fish need long-chain PUFA for optimal somatic growth and reproduction (Bell & Sargent, 2003; Ahlgren et al., 2009). Several feeding experiments have shown negative effects of LC-PUFA deficient diets on the growth rates of fish (Murray et al., 2014) or metabolic rates in birds (Twining et al., 2016) that may be related to the metabolic costs associated with converting dietary precursor PUFA (e.g., ALA or LA) to LC-PUFA. In late fall, the input of terrestrial insects to streams plays only a minor role in salmonid diets (Nakano & Murakami, 2001). Consequently, in this study only benthic invertebrates offered an optimal source of algal n-3 PUFA, in particular EPA (Ghioni et al., 1996), for further conversion, at levels that are sufficient for proper functioning in freshwater fish, even though past experimental studies have claimed low conversion rates (Buzzi et al., 1996). Previous research indicated a substantial contribution of algal carbon to consumer biomass is by stable isotope data, ranging from small tropical headwater streams (Lau et al., 2009) to large arid rivers (Bunn et al., 2013). Furthermore, isotopic evidence suggests most of the nitrogen assimilated by stream consumers is of algal origin, even in systems where there appears to be a significant terrestrial carbon contribution (Brett et al., 2017; Thorp & Bowes, 2017).

The high PUFA similarity between liver and muscle suggests that livers retained or endogenously transformed dietary PUFA and allocated them to muscles (Martin et al., 1994). Higher DHA levels in salmonid than in European bullhead livers could be the result of higher conversion rates or greater incorporation of dietary DHA from prey fish. However, given the fact that salmonids in this study were small and piscivory is not the primary mode of feeding for this size class (Elliott, 1973; Behnke et al., 2002), the high similarity between liver PUFA of European bullhead and both salmonid taxa (*S. trutta* and *O. mykiss*) implies that both were equally able to retain ALA and LA and further minimize potential dietary deficiencies in long-chain PUFA by endogenous conversion.

The ANOSIM results indicate that the PUFA composition in organs of both salmonid taxa (*S. trutta* and *O. mykiss*) were more similar to each other than to European bullhead. Gladyshev et al. (2018) reported that phylogeny (order identity) outweighs food or habitat as factors in fish muscle FA. In line with this argument, we argue that species identity could explain the high PUFA similarities between all sampled organs of Rainbow and Brown trout. Despite similar brain DHA content among both fish groups, the brain, and eyes of European bullhead contained more ARA than salmonids. This is also indicated by the more similar vector scores of DHA and ARA in the ordination plots of European bullhead than salmonids. Both ARA and EPA are associated with eicosanoid synthesis (e.g., prostaglandins) and act as hormone precursors (Sargent et al., 1995). Increased ARA content in brains and eyes of European bullhead could be related to spawning activities (Stacey & Goetz, 1982) as suggested by the presence of egg-bearing females observed during our sampling. Higher reproductive activities in European bullhead compared to salmonids are likely to have resulted in higher PUFA allocation and could explain higher similarities among organ PUFA. Changes in the FA composition due to reproductive activities were reported for gonadal, muscle, and liver tissues (Nogueira et al., 2017; Keva et al., 2019), but impacts on neural tissues have not been assessed yet. Other factors related to foraging behavior, eye anatomy or visual capabilities (Elliott, 1973; Mills & Mann, 1983; Guthrie, 1986; Wagner, 1990) could lead to differences in PUFA composition in brain and eye between European bullhead and salmonids, but further studies investigating such functional differences are still warranted.

The high dissimilarity in PUFA of salmonid brain and eyes was unexpected, but largely accounted for by differences in the lipid classes between these neural organs with direct implications for their PUFA composition: higher resemblance of dietary FA in eyes than brains (Bell & Dick, 1991; Tocher, 1993; Buzzi et al., 1996). This is consistent with other studies which showed that dietary inputs can significantly affect eye lipids (Bell et al., 1995; Brodtkorb et al., 1997; Navarro et al., 1997). Eyes store dietary FA for reasons that are still not fully understood. For example, apart from being major energy store, NL in eyes may serve as reservoir for membrane FA synthesis (Linares & Henderson, 1991). Similar to Brodtkorb et al. (1997), brain NL were composed predominantly of SAFA and when required, these could serve as energy source (De Roos, 1994) even if most energy is mobilized from glycogen in teleost brains (Soengas & Aldegunde, 2002). The high abundance of the monosaturated FA 18:1n-9 was reported in total lipids (Tocher & Harvie, 1988; Stoknes et al., 2004; Amlund et al., 2012) and the membrane lipids (Brodtkorb et al., 1997) of neural tissues (brain and eyes) of trout, salmon and marine fish species (Geurden et al., 1998). Farkas et al. (2000) suggested that high levels of 18:1n-9 and 22:6n-3 in fish membranes are required for proper fusion and signal transduction rates in cold-adapted animals. Although entire eyeballs were analyzed, the high similarity between PL of eyes and brain imply the provision of LC-PUFA to the retina may suffice even if basal diet sources in stream food webs are deficient in n-3 LC-PUFA. Such dietary shortage of n-3 LC-PUFA is, to varying degrees, compensated in stream fish via dietary PUFA conversion to target PUFA, such as DHA, that are required for neural organs. Consequently, the low dietary availability of DHA in the trophic base of these subalpine streams might not have impaired the visual acuity or proper functioning of sensory systems of fish.

Our large-scale field study provides further evidence that algal PUFA support fish in headwater stream habitats. Contrary to the implications of the River Continuum Concept, algae rather than terrestrial sources supplied aquatic vertebrates with high-quality biomolecules (e.g., PUFA) required for neurogenesis. Algal PUFA that were first incorporated by benthic invertebrates serve subsequently as precursors for critical PUFA required for fish organs, in particular for DHA in fish brains and eyes. Based on these data, it is not possible to differentiate whether DHA was allocated from livers to respective organs or if DHA production occurred locally, e.g., in brain astrocytes (Mourente et al., 1991; Bell et al., 2001; Tocher et al., 2006). Future efforts to elucidate trophic and allocation pathways of individual n-3 PUFA in fish organs and dietary sources require compound-specific stable isotope analyses of individual FA (Kühmayer et al., 2020).

## Electronic supplementary material

Below is the link to the electronic supplementary material.

Supplementary material 1 (DOCX 2524 kb)
