# Supplementary material for: Polyunsaturated fatty acids in fish tissues more closely resemble algal than terrestrial diet sources
Source: Hydrobiologia. 2020 Nov 16;848(2):371–83. doi: 10.1007/s10750-020-04445-1 (PMC7738338; doi:10.1007/s10750-020-04445-1)

# **Supplementary data**

**Online resource 1.**

Study area (Ybbs River catchment, 254km², 550- 1000 m.a.s.l) with 17 sampling sites (dots) at the 9 oligotrophic rivers (blue lines) of different stream orders (1-5). Bodingbach, BB (4), Faltlbach, FB (1), Kothbergbach oben, KBO (3), Kothbergbach unten, KBU (3); Lackenbach, LB (2); Oberer Seebach Lend, SL (2); Oberer Seebach Ritrodat, SR (2), Ois alte Säge, OS (4);Holzhüttenboden, HH (4); Schlechen, SCH (1); Tagles, TG (3); Taschlbach (1); Rehberghütte, RB (3), Weiße Ois, WO (3); Göstling Lagerhaus, YGL (5), Kläranlage Lunz, YKL (5), Lunz Großau, YLG (5).

**
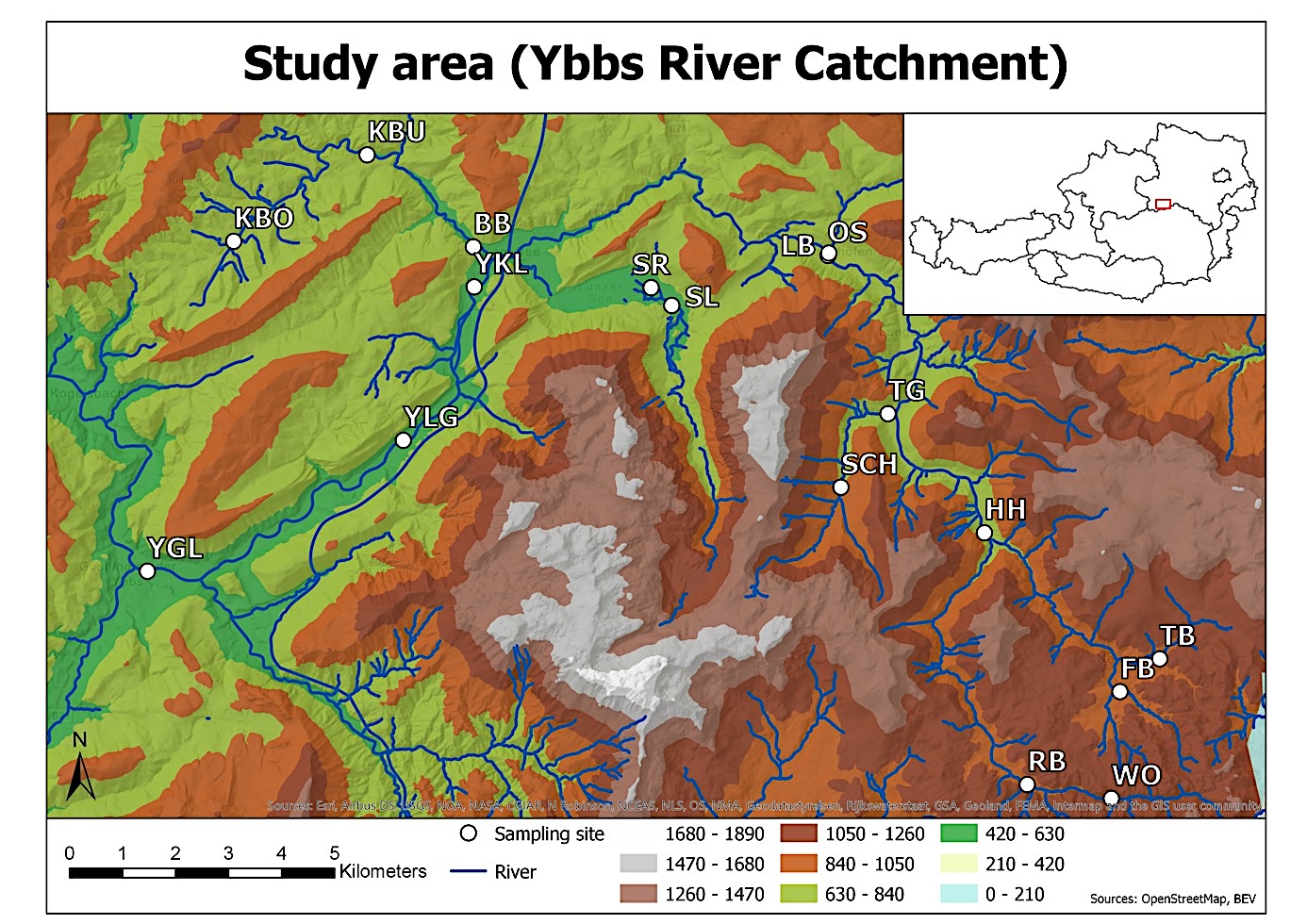
**

**Online resource 2.**

Mean body length (mm) and weight (g) of all three fish taxa (*Cottus gobio, Oncorhynchus mykiss, Salmo trutta fario*). n = sample size.

| **Taxa** | **n** | **Body length**  **[mm]** | **Body weight**  **[g]** |
| --- | --- | --- | --- |
| *Cottus gobio* | 64 | 90.9 ± 11.1 | 10.0 ± 4.1 |
| *Oncorhynchus mykiss* | 32 | 109.2 ± 35.2 | 17.5 ± 24.7 |
| *Salmo trutta fario* | 62 | 128.6 ± 49.5 | 28.9 ± 30.4 |

**Online resource 3.**

Lipid classes in salmonid brain and eyes after thin-layer-chromatography. Lipids in salmonid fish eyes contained neutral (NL) and polar (PL) lipids, brain lipids contained only polar lipids (PL).

**Online resource 4.**

Regression tree for exploring patterns based on untransformed percentage data of individual fatty acids (ALA, SDA, LA, EPA, ARA, DHA) among food-web components (epilithon, Conditioned and fresh leaves, benthic invertebrates, salmonid and European Bullhead tissues). Misclassification was <30%.


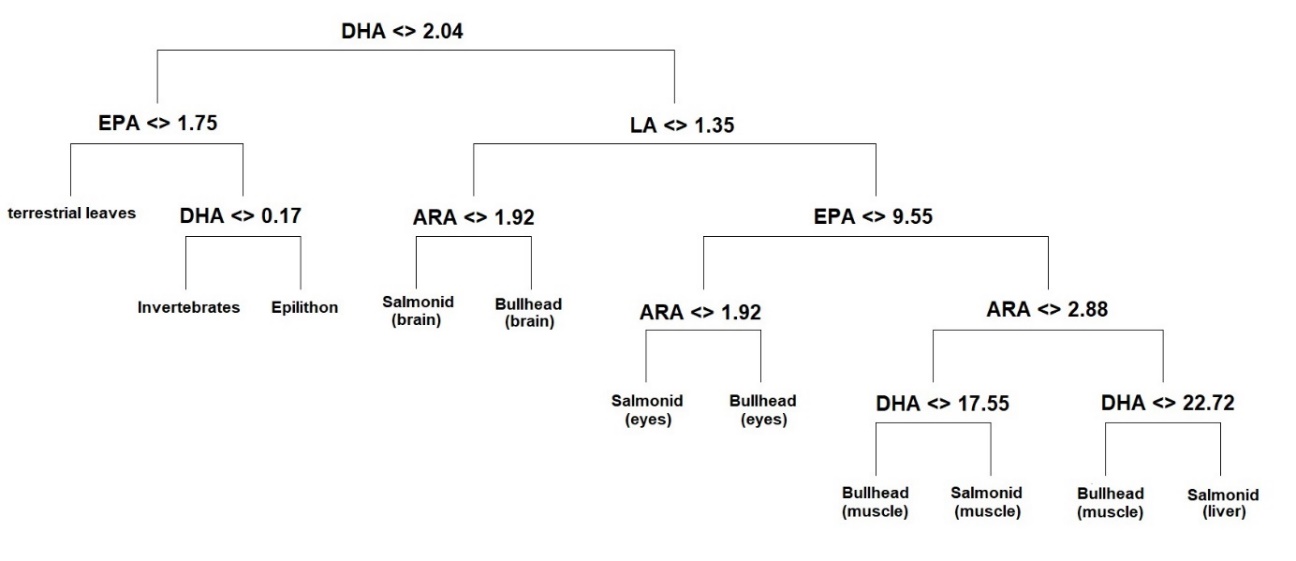


**Online resource 5.**

Mean (± 1 standard deviation mg g d w-1) polar and neutral lipid content of salmonid brain and eyes (n=70).


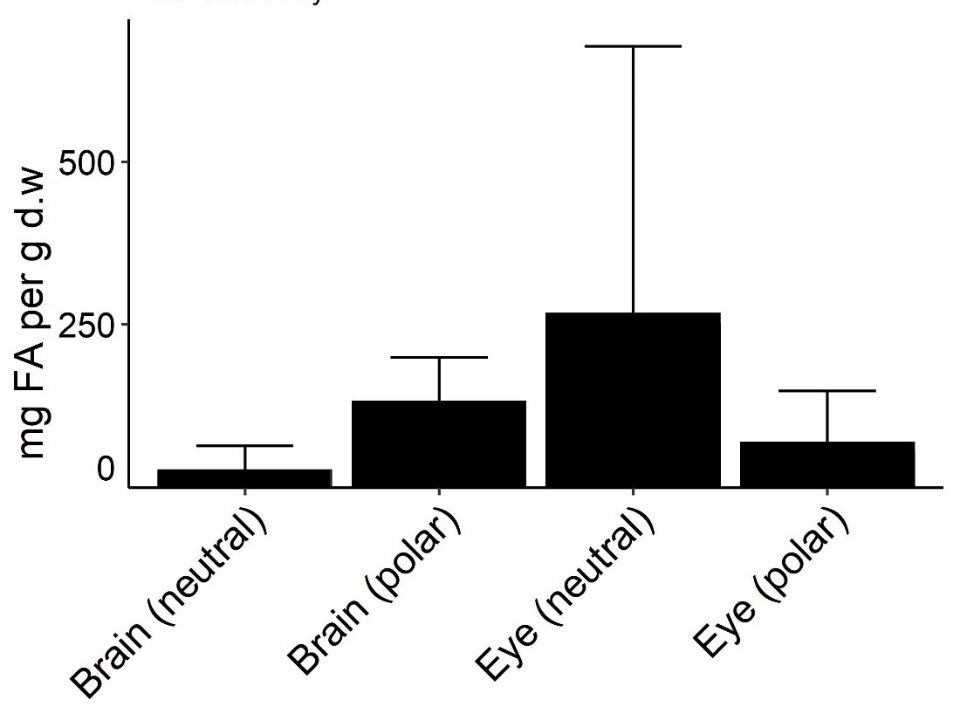

Supplement: Supplementary file 1 — Supplementary material 1 (DOCX 2524 kb) [file 10750_2020_4445_MOESM1_ESM.docx]
